# Draft genome sequencing and assembly of* Favolaschia claudopus* CIRM-BRFM 2984 isolated from oak limbs

**DOI:** 10.7150/jgen.92255

**Published:** 2024-02-17

**Authors:** David Navarro, Elodie Drula, Delphine Chaduli, Robert Cazenave, Steven Ahrendt, Jie Wang, Anna Lipzen, Chris Daum, Kerrie Barry, Igor V. Grigoriev, Anne Favel, Marie-Noëlle Rosso, Francis Martin

**Affiliations:** 1 INRAE, Aix Marseille Univ, UMR1163 Biodiversité et Biotechnologie Fongiques, 13288, Marseille, France.; 2 CIRM-CF, INRAE, Aix Marseille Univ, UMR 1163, 13009, Marseille, France.; 3 AFMB, CNRS, Aix Marseille Univ, UMR 7257, USC 1408, 13009, Marseille, France.; 4 Association Mycologique de Bigorre, 65600, Séméac, France.; 5 U.S. Department of Energy Joint Genome Institute, Lawrence Berkeley National Laboratory, Berkeley, CA 94720, USA.; 6 Department of Plant and Microbial Biology, University of California Berkeley, Berkeley, CA 94720, USA.; 7 INRAE, Univ de Lorraine, UMR 1136 Interactions Arbres/Microorganismes, 54280, Champenoux, France.; 8 Institute of Applied Mycology, College of Plant Science and Technology, Huazhong Agricultural University, Wuhan, Hubei 430070, China.

**Keywords:** Favolaschia, genome, Mycenaceae, decayed wood, Agaricales, Invasive species

## Abstract

*Favolaschia claudopus*, a wood-inhabiting basidiomycete of the Mycenaceae family, is considered an invasive species that has recently spread from Oceania to Europe. The CIRM-BRFM 2984 strain of this fungus was originally isolated from a basidiome collected from the fallen limb of a decayed oak tree in Southwest France. The genome sequence of this strain shared characteristics with other Mycenaceae species, including a large genome size and enriched content of protein-coding genes. The genome sequence provided here will facilitate further investigation on the factors that contribute to the successful global dissemination of *F. claudopus*.

## Introduction

The genus *Favolaschia* belongs to the Mycenaceae family within the order Agaricales. It is known for its poroid basidiomes that are found in dead plant materials worldwide. Previously considered as a variety of *Favolaschia calocera*, *F. claudopus* (Singer) Q.Y. Zhang & C. Dai has recently been elevated to the species status 1.* F. claudopus* is a wood-inhabiting fungus classified as an invasive species that has recently spread from Oceania to Europe 2; 3; 1. It is suspected that the introduction of this species may be favored by the presence of black locust (*Robinia pseudoacacia*), which is the primary host of *F. claudopus*. The adaptive capabilities of this species have raised concerns among mycologists as they could potentially endanger local fungal species 4; 5. The first specimens of *F. claudopus* in metropolitan France were observed in Pyrénées-Atlantiques (southwest France). We have carried out genome sequencing of the* F. claudopus* strain CIRM-BRFM 2984 as part of the 1000 Fungal Genomes (1KFG) project, an extensive international research initiative focused on sequencing the genomes of various fungal taxa, in collaboration with the Joint Genome Institute of the U.S. Department of Energy 6.

**Figure 1:**
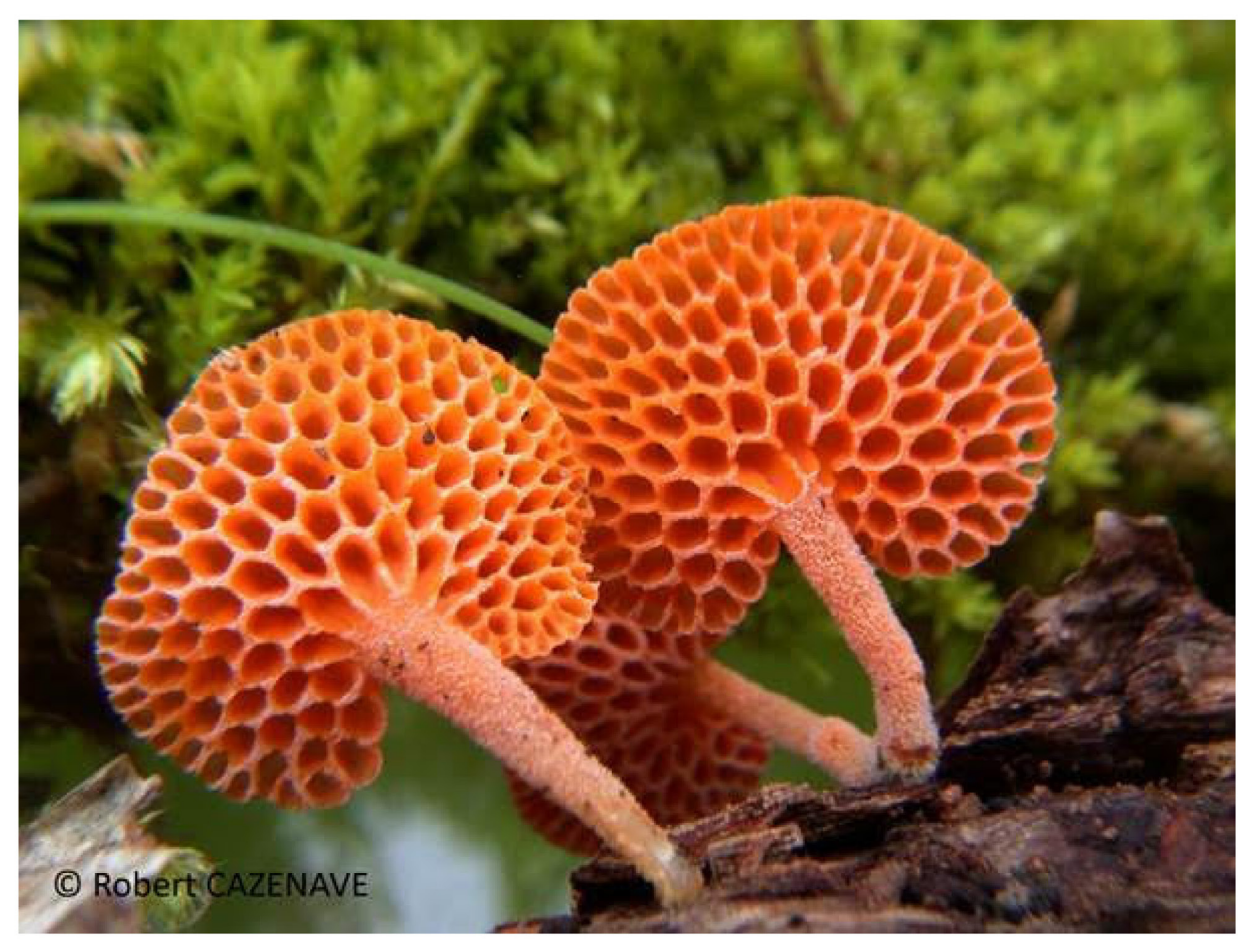
Favolaschia claudopus basidiomes.

CIRM-BRFM 2984, a strain of *F. claudopus*, was isolated from a basidiome collected from the fallen branch of a decayed *Quercus* tree in Agnos, France, in November 2018 (refer to Fig. 1 for visual representation). The basidiome was dried and preserved using voucher number AMB49273. The isolation process involved transferring tissues from the basidiome to agar plates containing malt extract supplemented with antibiotics (0.025% chloramphenicol and 0.04% gentamicin). Successive subcultures without antibiotics were performed to verify the absence of contaminants. To confirm the identity of the isolated strain, the rDNA barcoding region ITS1-5,8S-ITS2 was PCR-amplified and sequenced, as previously described 7. Following molecular authentication, the strain was deposited in the Biological Resource Center CIRM-CF (International Center of Microbial Resources, Marseille, France; https://doi.org/10.15454/KJQW-SJ57; www.cirm-fungi.fr) under the accession number CIRM-BRFM 2984.

Genomic DNA was extracted from the ground mycelia according to the protocol described by Lomascolo et *al.* 8. In order to enhance gene structural annotation, we sequenced the mRNAs obtained after a 3-day growth period on three different media: **A**: Glucose 10 g.L-1, bactopeptone 5 g.L-1, yeast extract 2.5 g.L^-1^; **B**: Malt extract 15 g.L^-1^, salts (KH_2_PO_4_ 0.2 g.L^-1^, CaCl_2_·2H_2_O 1.32.10^-2^ g.L^-1^, MgSO_4_·7H2O 0.5 g.L^-1^, FeSO_4_·7H_2_O 0.07 g.L^-1^, ZnSO_4_·7H_2_O 7.77.10^-3^ g.L^-1^, MnSO_4_·H_2_O 3.63.10^-3^ g.L^-1^, CuSO_4_·5H_2_O 7.2.10^-4^ g.L^-1^) and thiamine 250.10^-3^ g.L^-1^; **C**: Maltose 10 g.L^-1^, yeast extract 2.5 g.L^-1^, salts and thiamine 250.10^-3^ g.L^-1^. Fresh mycelia were harvested, pooled, and ground in liquid nitrogen using a cryogenic grinder (SPEX Sample Prep; UK). Subsequently, total RNAs were extracted from 100 mg of ground tissue using TRIZOL (Ambion), followed by precipitation with isopropanol, resuspension in water, and treatment with RNase-free Dnase I (QIAGEN). Total RNAs were then precipitated with LiCl and resuspended in DEPC-treated water according to the protocol described by Miyauchi et *al.* 9.

**Figure 2:**
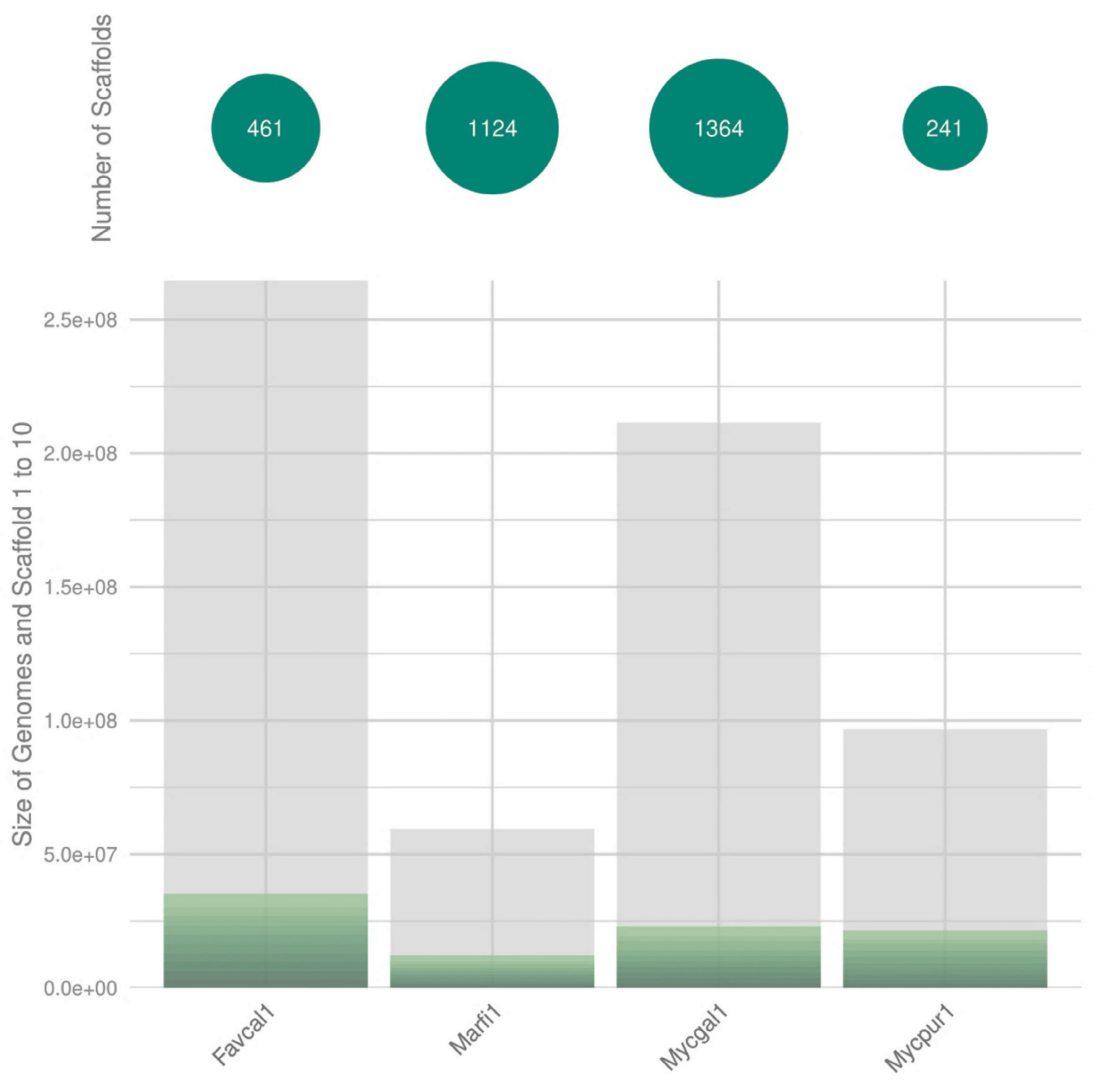
Genome sequence assembly. The size of the 10 largest scaffolds (green sections) from *F. claudopus* CIRM-BRFM 2984 v1.0 (Favcal1_2) was compared with that of the genomes available for the related species* Marasmius fiardii* PR-910 v1.0 (Marfi1), *Mycena galopus* ATCC-62051 v1.0 (Mycgal1) and *Mycena pura* (Mycpur1).

The *F. claudopus* CIRM-BRFM 2984 v1.0 genome was sequenced from 162 µg of genomic DNA using the Pacific Biosciences sequencing platform (>10kb PacBio libraries with Blue Pippin size selection), assembled with Falcon v. 0.0.8 10, polished with Arrow version SMRTLINK v8.0.0.80529 (https://www.pacb.com/support/software-downloads), and annotated with the JGI Annotation Pipeline 6. mRNA sequences were obtained using Illumina RNA-Seq data assembled with Trinity v2.11.0 11. The genome size of* F. claudopus* (265 Mbp) was similar to that of other strains of the Mycenaceae family 9. The draft genome assembly (Table 1) was significantly improved compared to that of previously sequenced *Mycena* species, with the 10 largest scaffolds covering 13.3% of the genome (Fig. 2). We used BUSCO (BUSCO v3.0.2) with default parameters and the agaricales_odb10 database 12 to benchmark universal single-copy orthologues and assess the completeness of the genome *Favolaschia claudopus* CIRM-BRFM 2984 v1.0). The completeness of these genome assembly was compared to that of three Mycenaceae genomes; *Marasmius fiardii* PR-910 v1.0, *Mycena galopus* ATCC-62051 v1.0 and *Mycena pura* 9144 v1.0. The BUSCO scores varied from 87.4% to 92.8%. Regarding the genome of *Favolaschia claudopus*, the completeness was the highest (92.8%) and only 6.4% of BUSCO genes were missing. It should be noticed that 86.8% (3358) of the BUSCO genes were identified as duplicated sequences. After annotation of the protein-coding genes, we identified a large repertoire of CAZymes (1,340 genes), peptidases (891 genes), and transporters (1,592 genes).

Further research, including comparative genomic analysis with other *Mycena* species, will aid in determining whether the expansive genome of* F. claudopus* has facilitated its ability to disperse and establish itself in diverse ecological niches.

## Figures and Tables

**Table 1:** Genome assembly information.

Genome Assembly size	264.58Mbp
Sequencing read coverage depth	20.47x
# of contigs	461
Contig N50/L50	61/1.46Mbp
# of gene models	49,883
